# Frequency-Selective Surface-Based MIMO Antenna Array for 5G Millimeter-Wave Applications

**DOI:** 10.3390/s23157009

**Published:** 2023-08-07

**Authors:** Iftikhar Ud Din, Mohammad Alibakhshikenari, Bal S. Virdee, Renu Karthick Rajaguru Jayanthi, Sadiq Ullah, Salahuddin Khan, Chan Hwang See, Lukasz Golunski, Slawomir Koziel

**Affiliations:** 1 Telecommunication Engineering Department, University of Engineering and Technology, Mardan 23200, Pakistan; iftikharuddin114@gmail.com (I.U.D.); sadiqullah@uetmardan.edu.pk (S.U.); 2 Department of Signal Theory and Communications, Universidad Carlos III de Madrid, 28911 Leganés, Madrid, Spain; 3 Center for Communications Technology, London Metropolitan University, London N7 8DB, UK; b.virdee@londonmet.ac.uk (B.S.V.); r.k.r.jayanthi@londonmet.ac.uk (R.K.R.J.); 4 College of Engineering, King Saud University, P.O. Box 800, Riyadh 11421, Saudi Arabia; drskhan@ksu.edu.sa; 5 School of Computing, Engineering and the Built Environment, Edinburgh Napier University, 10 Colinton Rd., Edinburgh EH10 5DT, UK; c.see@napier.ac.uk; 6 Faculty of Electronics, Telecommunications and Informatics, Gdansk University of Technology, 80-233 Gdansk, Poland; 7 Engineering Optimization & Modeling Center, Reykjavik University, 101 Reykjavik, Iceland

**Keywords:** MIMO antenna, millimeter-wave (mm wave) region, wide bandwidth, mutual coupling reduction, frequency-selective surface, fifth generation (5G)

## Abstract

In this paper, a radiating element consisting of a modified circular patch is proposed for MIMO arrays for 5G millimeter-wave applications. The radiating elements in the proposed 2 × 2 MIMO antenna array are orthogonally configured relative to each other to mitigate mutual coupling that would otherwise degrade the performance of the MIMO system. The MIMO array was fabricated on Rogers RT/Duroid high-frequency substrate with a dielectric constant of 2.2, a thickness of 0.8 mm, and a loss tangent of 0.0009. The individual antenna in the array has a measured impedance bandwidth of 1.6 GHz from 27.25 to 28.85 GHz for S_11_ ≤ −10 dB, and the MIMO array has a gain of 7.2 dBi at 28 GHz with inter radiator isolation greater than 26 dB. The gain of the MIMO array was increased by introducing frequency-selective surface (FSS) consisting of 7 × 7 array of unit cells comprising rectangular C-shaped resonators, with one embedded inside the other with a central crisscross slotted patch. With the FSS, the gain of the MIMO array increased to 8.6 dBi at 28 GHz. The radiation from the array is directional and perpendicular to the plain of the MIMO array. Owing to the low coupling between the radiating elements in the MIMO array, its Envelope Correlation Coefficient (ECC) is less than 0.002, and its diversity gain (DG) is better than 9.99 dB in the 5G operating band centered at 28 GHz between 26.5 GHz and 29.5 GHz.

## 1. Introduction

The development of wireless communication networks has indeed been a significant modern uprising in the telecom industry. Wireless communication allows for the transmission of voice, data, and multimedia over the airwaves without the need for physical wired connections [1]. Between 2015 and 2020, global mobile data traffic saw a significant surge. According to various reports, the compound annual growth rate (CAGR) of mobile data traffic during this period was around 45%. This exponential increase was primarily driven by the growing number of smartphones, tablets, and other connected devices, as well as the rise of mobile applications and video streaming services. To meet the growing demand for wireless data, regulatory bodies and industry stakeholders continually work on allocating additional spectrum resources. Spectrum auctions and reallocations are conducted to ensure that a sufficient spectrum is available for mobile communication operators to deploy and expand their networks. To meet the data requirement challenges of wireless communications, 5G technology offers lower latency and improved spectral efficiency compared to previous generations. It employs advanced techniques such as massive multiple-input and multiple-output (MIMO), beamforming, and dynamic spectrum sharing to enhance capacity and improve spectral efficiency [2]. 

The 5G networks utilize both sub-6 GHz and millimeter-wave spectrum to provide enhanced performance and capabilities. Sub-6 GHz spectrum refers to the frequency bands below 6 GHz, including bands such as 600 MHz, 700 MHz, 2.5 GHz, 3.5 GHz, and 5 GHz [3,4]. The sub-6 GHz spectrum offers better coverage and penetration through buildings and obstacles compared to higher-frequency bands. It is well suited for providing wide-area coverage and supporting applications that require broader coverage, such as mobile broadband services. The millimeter-wave spectrum operates in higher-frequency bands, typically between 24 GHz and 100 GHz [5,6,7,8,9]. These frequencies offer significantly higher data transfer rates but have a limited range and are more susceptible to signal attenuation from obstacles like buildings and foliage. The millimeter spectrum is particularly well suited for providing high-capacity and low-latency connections in dense urban areas and specific hotspot locations. It enables the delivery of ultra-fast speeds and supports bandwidth-demanding applications such as virtual reality, augmented reality, and high-definition video streaming. However, millimeter-wave transmissions are affected by atmospheric attenuation, such as fog, rain, and snow. To overcome the challenges posed by atmospheric attenuation in millimeter-wave communications, high gain and high directive antennas are often used. These antennas focus the transmitted signal in a specific direction, increasing the signal strength and improving the overall link quality. 

Multiple-input multiple-output (MIMO) antenna systems play a vital role in modern and upcoming mobile communication technologies. MIMO technology enables the use of multiple antennas at both the transmitter and receiver, working simultaneously to improve link reliability, enhance channel capacity, and achieve higher throughput in terms of gigabits per second (Gbps) [10,11,12]. The design of MIMO antenna arrays comes with several challenges that need to be addressed to ensure optimal performance and reliability. Some of the key challenges in designing MIMO antenna arrays include reduction in mutual coupling, the spatial arrangement of radiating elements, operational bandwidth, complexity and cost, physical size, and form factor. Mutual coupling occurs when the presence of one antenna affects the performance of other nearby antennas in the array. This coupling can lead to interference between antennas, reducing the overall system performance. Managing mutual coupling is crucial in MIMO antenna design to maintain antenna isolation and minimize the impact on signal quality. The spatial arrangement and placement of antenna elements in the array can significantly affect the performance of the MIMO system. Determining the optimal placement to achieve desired radiation patterns, coverage, and mutual coupling reduction is a complex task that requires careful analysis and optimization. MIMO antenna arrays need to operate across multiple frequency bands or wide bandwidths to support various communication standards and requirements. Ensuring consistent performance and impedance matching over a broad frequency range is a challenge in MIMO antenna design. As the number of antennas in the array increases, so does the complexity and cost of the MIMO system. Each antenna element requires its own RF chain, which adds complexity to the system design and increases hardware costs. Managing the complexity and cost while achieving the desired performance is a challenge in MIMO antenna array design. The physical size and form factor of MIMO antenna arrays are essential considerations, especially in mobile devices or small form factor applications. Balancing the desired antenna performance with space limitations can be a challenge, requiring innovative design techniques such as compact antenna structures and integration with other components.

The authors in [9] reported a four-element MIMO antenna array operating at 28 GHz with a gain of 5.42 dBi. The MIMO has an overall size 30 × 30 × 1.575 mm^3^. In [13], the authors reported a 5G MIMO array that has a maximum gain of 7.1 dBi at 27 GHz and operates across 25.5–29.6 GHz. The MIMO antenna in [14] is shown to achieve a maximum gain of 8.3 dBi with a low isolation of 17 dB. Reported in [8] is a planar 28 GHz MIMO antenna inspired by a helix structure. The MIMO antenna has an impedance bandwidth of 3.89 GHz between 26.25 and 30.14 GHz, with an end-fire radiation pattern that has a maximum measured gain of 5.83 dBi. In [15], the authors reported a 28 GHz MIMO antenna for 5G applications where the radiating elements consists of conjoined triplet circular-shaped rings overlapped by conjoined two larger circular-shaped rings. The MIMOI array has a measured gain of 5.5 dBi. Reported in [16] is a 4 × 4 MIMO Dielectric Resonator Antenna (DRA) for 5G applications. DRA has an operating range from 26.71 GHz to 28.91 GHz with an isolation of 29 dB at 28 GHz, and a maximum gain of 7 dBi. The MIMO antenna reported in [17] has a Defected Ground Structure (DGS). The MIMO antenna is designed at 26.414 GHz and has a −6 dB bandwidth of 3.5346 GHz. The maximum gain of the MIMO antenna is limited to 6.22 dBi. Described in [18] is a two-element MIMO antenna for millimeter-wave applications at 28 GHz. The MIMO elements are arranged parallel to each other and placed between the elements is a metamaterial slab, which is shown to improve inter element isolation by 64 dB at 28 GHz. The maximum gain of the MIMO antenna is 8.75 dB at 28 GHz. 

This paper describes the results of an investigation of improving the gain and inter radiation element isolation of a 2 × 2 MIMO antenna array for 5G millimeter-wave applications. To minimize the mutual coupling between the radiating elements in the MIMO array, the radiating elements are spatially arranged to be orthogonal with respect to each other. The proposed MIMO array has an impedance bandwidth of 1.6 GHz, a gain of 7.2 dBi, and an inter radiator isolation greater than 26 dB. It is shown here that the gain of the MIMO array can be enhanced by placing a frequency-selective surface (FSS) over the array. With the FSS, the gain achieved at 28 GHz is 8.6 dBi. The planar MIMO antenna array is compact and of a simple construction that facilitates easy integration in 5G wireless communication systems. 

## 2. Antenna Design Procedure

**Figure 1:**
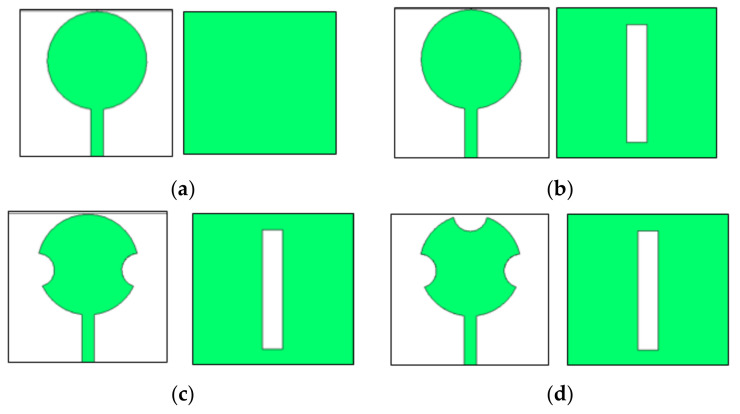
Evolution of the proposed radiating patch antenna. (**a**) Step #1. (**b**) Step #2. (**c**) Step #3. (**d**) Step #4.

**Figure 2:**
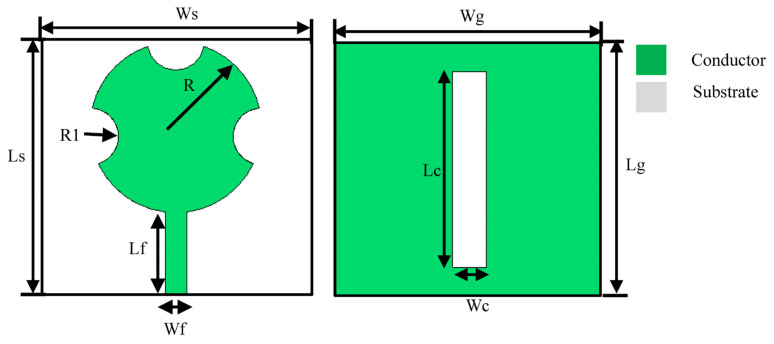
Front and back view of the proposed antenna.

**Figure 3:**
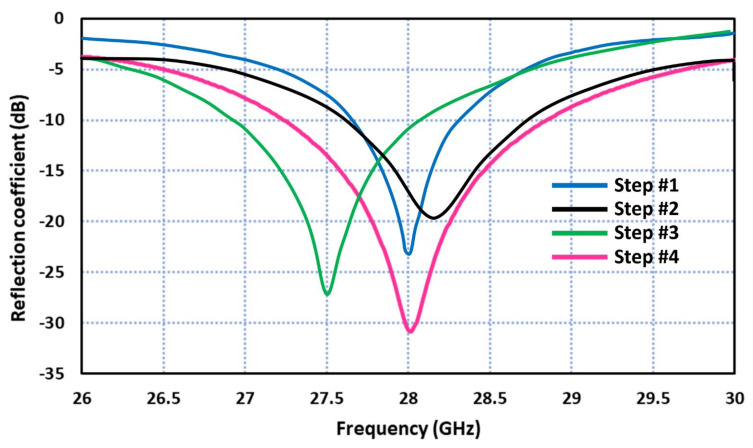
Reflection coefficient response of the modified circular patch antenna.

The antenna configuration is based on a standard circular patch antenna that is edge-fed. The circular antenna is modified through four steps, depicted in Figure 1. In the first step, the circular patch antenna is designed. The initial radius (R) of the circular patch antenna in cm can be calculated using the following expression [19].

(1) $$R=\frac{F}{\sqrt{1+\frac{2h}{\pi \varepsilon_{r}F}\left(ln\left(\frac{\pi F}{2h}\right)+1.7726\right)}}$$

where $F=8.791\times 10^{9}/f\sqrt{\varepsilon_{r}}$, h is the height of the substrate in cm, $\varepsilon_{r}$ is the dielectric constant of the substrate, and f is the resonant frequency of the patch in Hz. The initial circular patch antenna was designed at 28 GHz based on Equation (1) using Roger’s RT/Duroid 5880 substrate with a dielectric constant of 2.2, a thickness of 0.8 mm, and a loss tangent of 0.0009. The overall antenna size is 18 × 19 × 0.8 mm^3^. Figure 2 shows the physical parameters defining the antenna. Figure 3 shows the simulated reflection coefficient response of the antenna corresponding to each step using CST Microwave Studio. The presence of a ground plane rectangular slot improves the impedance bandwidth of the patch antenna as it introduces additional resonant modes. Semicircular sections are cut out of the circular patch to center the resonant frequency at 28 GHz. The antenna resonates at the prescribed frequency of 28 GHz and has an impedance bandwidth of 1.6 GHz from 27.25 to 28.85 GHz for S_11_ ≤ −10 dB. The physical parameter dimensions of the antenna structure are listed in Table 1.

## 3. Effect of Ground Plane Rectangular Slot

**Figure 4:**
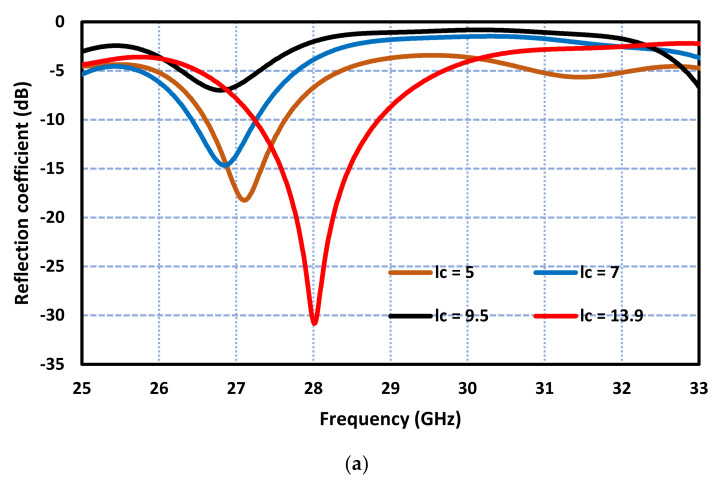

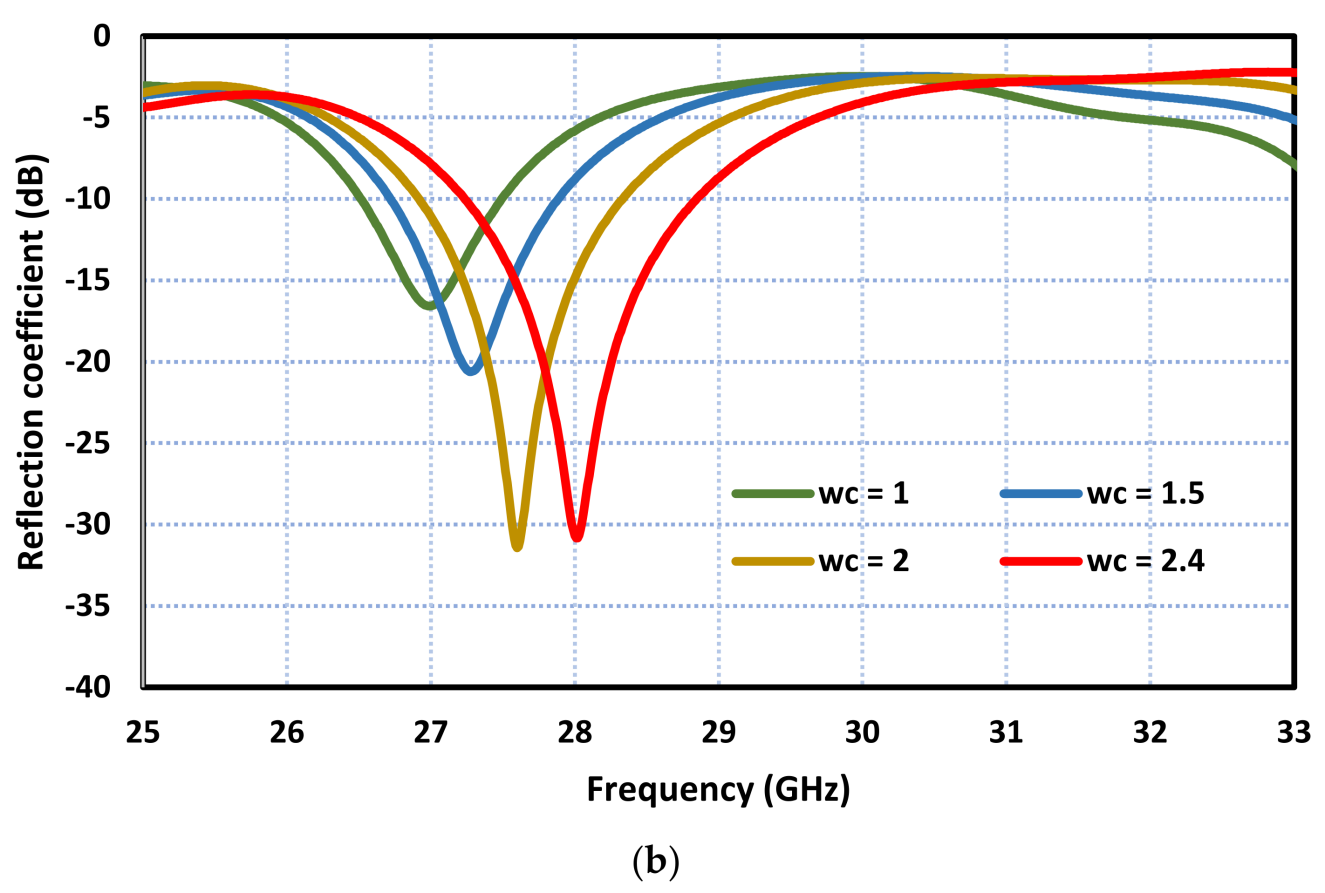
The effect on the reflection coefficient response of the antenna (**a**) by the slot length (Lc) and (**b**) by the slot width. Units of the length and width are in millimeters.

The effect of the ground plane rectangular slot on the antenna’s performance was analyzed in detail. Figure 4 shows how the dimensions of the slot affect the performance of the antenna. The parametric study shows that as the length of the slot is decreased, the resonance frequency of the antenna decreases, and the impedance bandwidth deteriorates significantly. A decrease in the slot width from 2.4 mm to 2 mm decreases the resonance frequency by 400 MHz with virtually no effect on the impedance bandwidth of the antenna. This property can be used to finetune the resonance frequency of the antenna. However, a further decrease in the width causes the impedance bandwidth to deteriorate significantly. The salient performance parameters of the antenna for various slot lengths and widths are listed in Table 2. The widest impedance bandwidth of 2 GHz is obtained for a slot length and width of 13.9 mm and 2.4 mm, respectively.

## 4. MIMO Antenna Array

**Figure 5:**
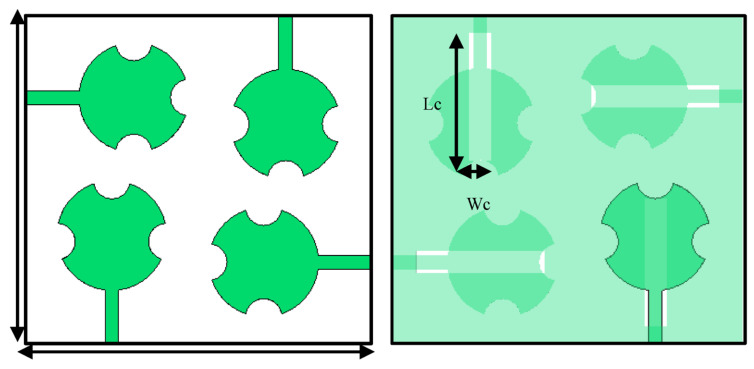
Front and back view of the proposed 2 × 2 MIMO antenna array.

**Figure 6:**
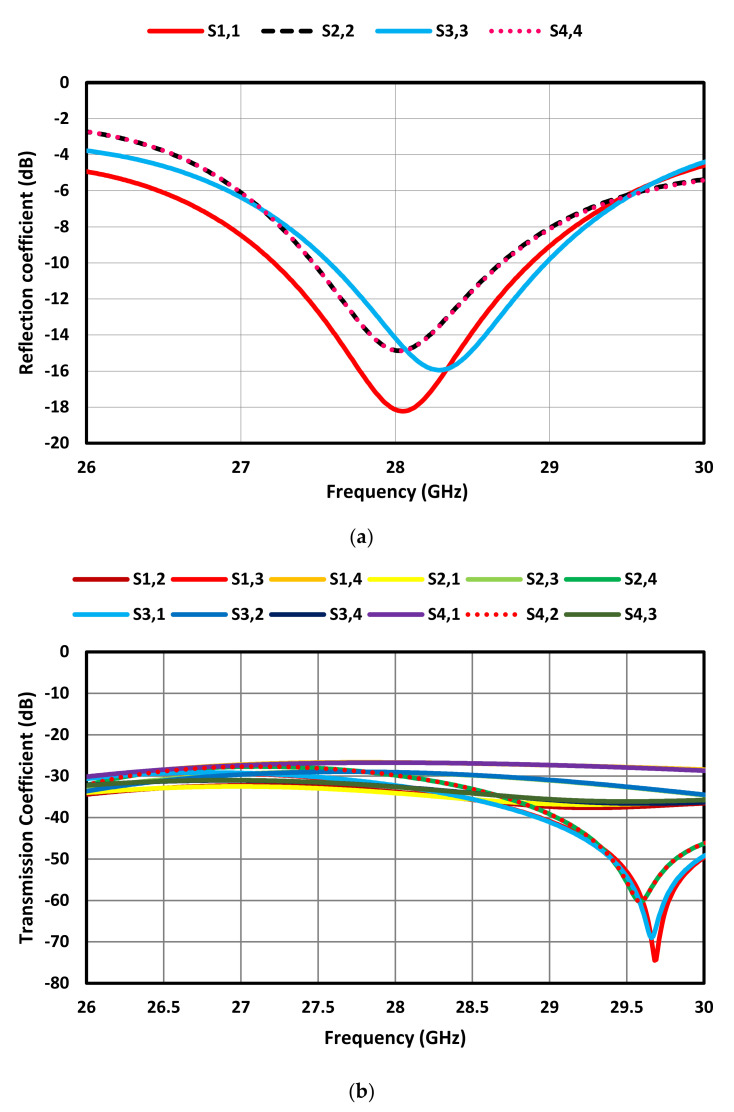
S-parameters of the proposed MIMO antenna array. (**a**) Reflection coefficient and (**b**) Transmission coefficient.

The modified circular patch antenna was used in the design of a 2 × 2 MIMO antenna array operating at 28 GHz. The individual antennas in the array were spatially arranged orthogonally with respect to each other, as shown in Figure 5 to improve the isolation between the individual antennas. This configuration also has the benefit of creating circular polarization. The MIMO antenna array was fabricated on Roger’s RT/Duroid 5880 substrate with a dielectric constant of 2.2, a thickness of 0.8 mm, and a loss tangent of 0.0009. The overall dimension of the MIMO is 38 × 36 × 0.8 mm^3^. The simulation results in Figure 6 show that at 28 GHz, the impedance matching at all four ports is better than −14 dB and the isolation between the radiating elements is better than 26 dB. 

## 5. Frequency-Selective Surface Unit Cell

It is shown here that by locating the proposed frequency-selective surface (FSS), the proposed MIMO antenna array can reduce mutual coupling between the radiating elements, resulting in improved gain performance. This is because in an antenna array, mutual coupling occurs when the electromagnetic fields from one antenna interact with other antennas in the array. This interaction can lead to changes in the radiation pattern, impedance, and efficiency of the antennas, degrading the overall performance of the array. The FSS is used here as a bandpass structure, allowing signals to pass through the frequency range of interest and isolating the interaction of the antennas outside their frequency band. When electromagnetic (EM) waves incident on the FSS structure, they incite electric currents into the array elements. The level of coupling energy defines the magnitude of the produced currents. The generated currents also work as EM sources, and they create additional scattered fields. Incident EM fields combined with these scattered fields make up the resultant field in the surrounding of FSS. The operational theory of FSS-based structures has been explained by Munk in detail [20].

**Figure 7:**
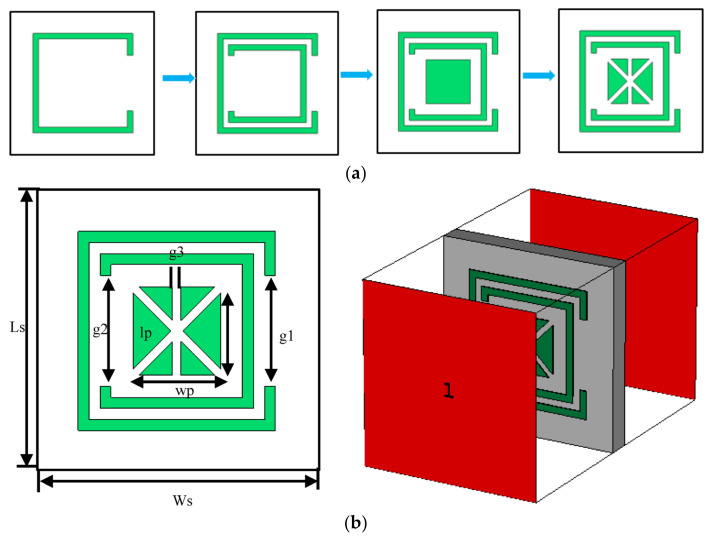

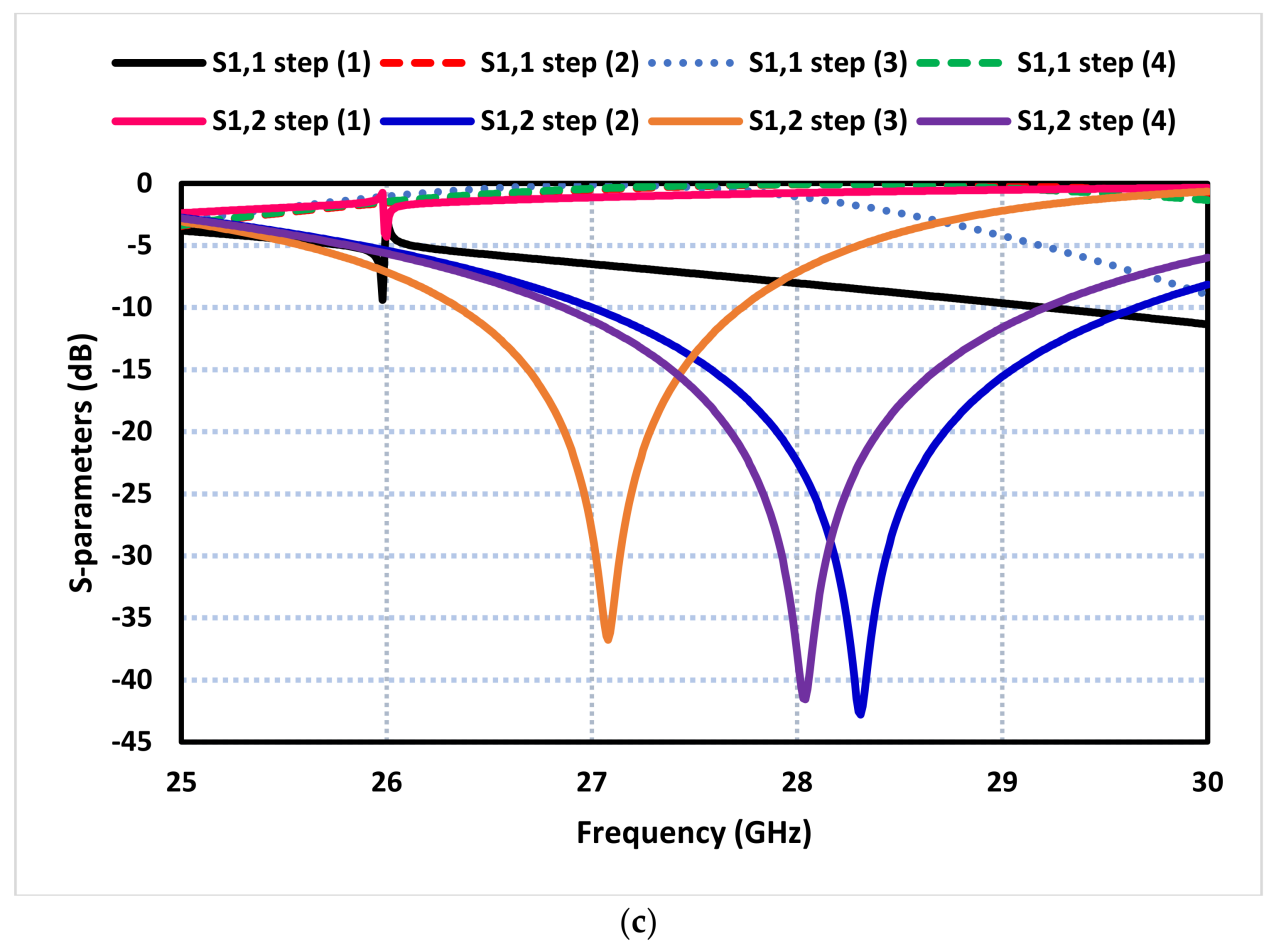
(**a**) Steps taken to create the proposed FSS unit cell. (**b**) Parameters defining the FSS unit cell and simulation excitation ports, and (**c**) S-parameter responses of the proposed FSS unit cell.

The steps taken to design the FSS unit cell are shown in Figure 7. The design starts with a C-shaped resonator. In the next step, a smaller C-shaped resonator is inserted inside the bigger C-shaped structure but facing the opposite direction. In the next step, a square patch is embedded inside the structure. In the final step, the central patch is divided with diagonal and vertical slots, as shown in Figure 7b. The dimensions of the FSS structure as defined in Figure 7b are listed in Table 3. Figure 7b also shows the boundary condition and port excitation used in CST Microwave Studio. The simulated reflection and transmission coefficient responses corresponding to each step are shown in Figure 7c. Step 4 of the FSS shows the impedance bandwidth for S_12_ ≤ −10 dB is 2.3 GHz from 26.9 GHz to 29.2 GHz.

## 6. FSS-Loaded Antenna

**Figure 8:**
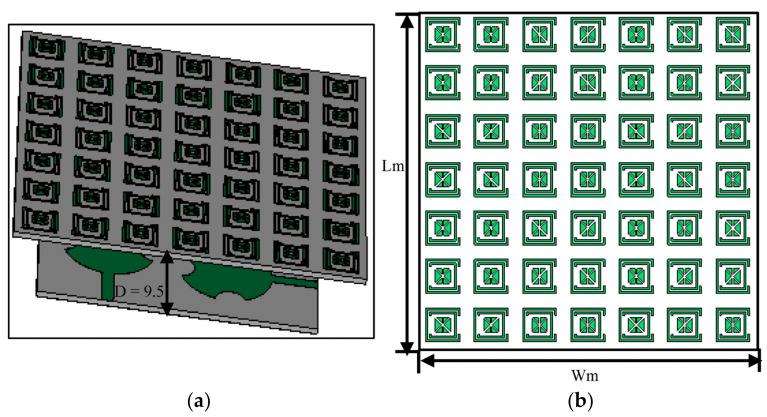
(**a**) FSS array surface located under the MIMO antenna array, and (**b**) FSS reflector.

The FSS, consisting of a 7 × 7 array of unit cell matrix, was used to enhance the performance of the antenna. This was achieved by locating the FSS structure over the MIMO antenna. The FSS structure allowed EM radiation to pass through that was within the operational frequency range of the MIMO antenna. The FSS structure was designed to have a bandpass between 27 GHz and 29 GHz. Figure 8 illustrates the mounting of the FSS with the proposed MIMO antenna. The gap between the FSS structure and the MIMO antenna was made to be an integer multiple of λg/2 for the reflected radiation to interfere constructively with the forward radiated waves. The FSS was fabricated on Roger’s RT/Duroid 5880 substrate with a dielectric constant of 2.2, a thickness of 0.8 mm, and a loss tangent of 0.0009. The footprint of the FSS array is 45 × 45 mm^2^. 

**Figure 9:**
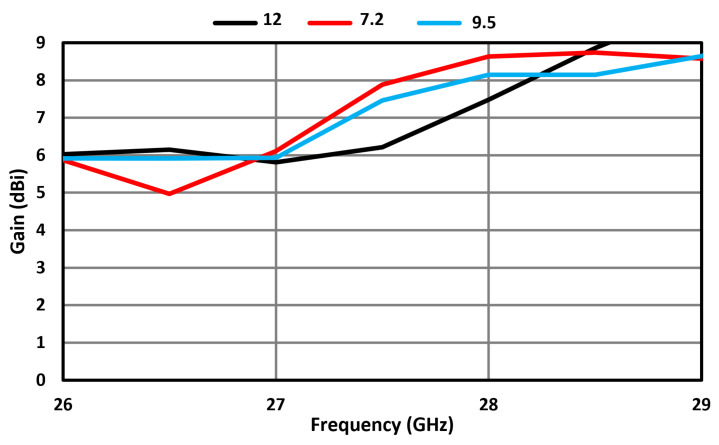
Gain of FSS-based MIMO antenna array at different gaps. Units are in millimeters.

**Figure 10:**
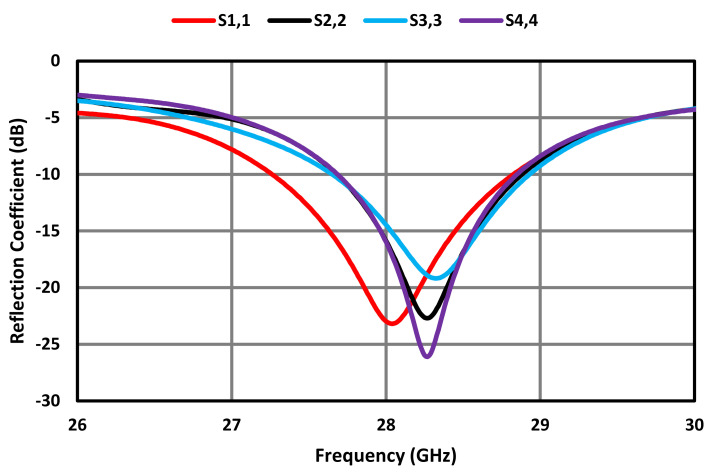
Reflection coefficient at the four ports of the FSS-based MIMO antenna array.

Figure 9 shows how the gain performance of the MIMO antenna was affected for various gaps between the FSS surface and the proposed MIMO antenna. At a gap of 7.2 mm corresponding to λg/2, the gain achieved at 28 GHz was 8.65 dBi; however, at a gap of 9.5 mm and 12 mm, the gain at 28 GHz was 8.1 dBi and 7.4 dBi, respectively. Therefore, a gap of 7.2 mm was used in the design of the MIMO antenna. The reflection coefficient at the four ports of the FSS-based MIMO antenna array is shown in Figure 10. It can be observed that the MIMO antenna array resonates at 28 GHz and its operational bandwidth for S_11_ ≤ −10 dB is within the 5G mm wave spectrum between 26.5 GHz and 29.5 GHz. 

**Figure 11:**
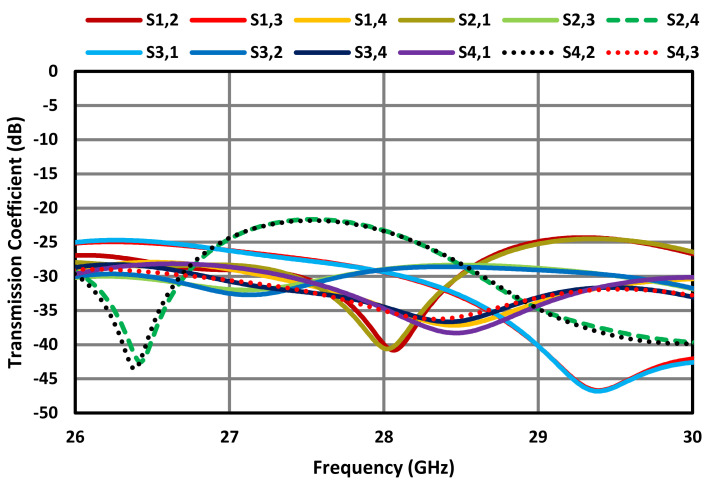
Transmission coefficients of the FSS-based MIMO antenna array.

Figure 11 shows that the orthogonal arrangement of individual antennas in the proposed FSS-based MIMO array improves isolation between the radiating elements, which is better than 20 dB across a wide frequency range between 26 GHz and 30 GHz. This is because the correlation between the antennas is reduced. The correlation between antennas in an MIMO system is a measure of the similarity of their received signals. A high correlation between antennas can limit the system’s ability to exploit the spatial diversity offered by MIMO, leading to a decrease in the achievable capacity gain. When the antennas are closely spaced or placed in a non-orthogonal configuration, they tend to experience higher mutual coupling and correlations. This is due to the unwanted electromagnetic interaction between adjacent antennas in the array. This coupling can result in signal interference, reduced antenna efficiency, and increased sensitivity to changes in the propagation environment.

## 7. Measured Results

**Figure 12:**
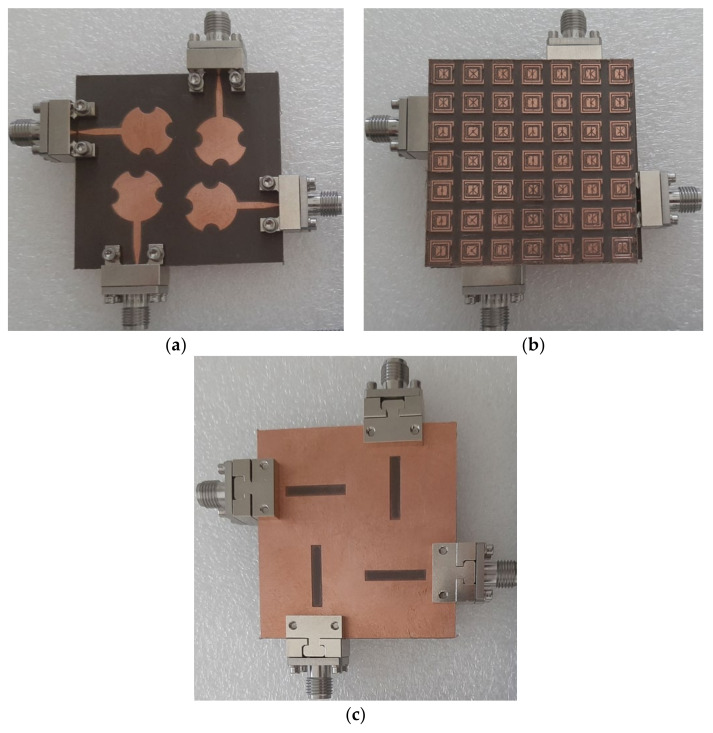
Fabricated prototype of the FSS-based MIMO antenna array, (**a**) front view, (**b**) FSS layer, and (**c**) back side view.

The fabricated prototype of an FSS-based MIMO antenna array is shown in Figure 12. The proposed MIMO array was constructed on Roger’s RT/Duroid 5880 substrate with a dielectric constant of 2.2, a thickness of 0.8 mm, and a loss tangent of 0.0009. The gap between the ground plane of the MIMO array and the FSS was 7.2 mm for the high gain performance at 28 GHz, as determined in Section 6. It was ensured that the power level and the phase of the excitation signals at the four ports were identical. The measured and simulated results are discussed in the subsequent sub-sections.

### 7.1. S-Parameters

**Figure 13:**
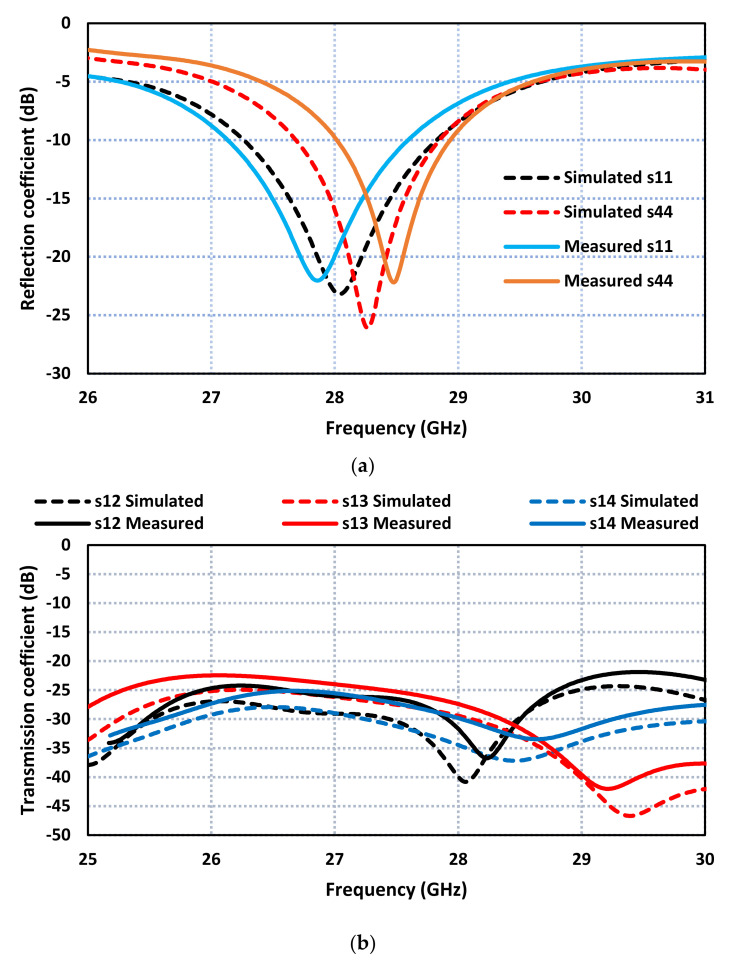
The measured and simulated results of the FSS-based MIMO antenna array. (**a**) Reflection coefficient response. (**b**) Transmission coefficient response.

The simulated and measured S-parameters of the FSS-based MIMO antenna are compared in Figure 13. The simulated impedance bandwidth, shown in Figure 13a, at port 1 is 1.65 GHz from 27.2 to 28.85 GHz, and at port 4 is 1.15 GHz from 27.7 GHz to 28.85 GHz. The measured impedance bandwidth at port 1 is 1.5 GHz from 27.1 to 28.6 GHz, and at port 4 is 0.95 GHz from 28 GHz to 28.95 GHz. The measured isolation between the four ports in Figure 13b is better than 20 dB. The discrepancy between the measured and simulated results of the impedance bandwidth and isolation between the radiating elements in the proposed MIMO antenna is attributed to several factors. These factors include manufacturing tolerances, unwanted antenna coupling, and inaccurate simulation models. Although an orthogonal arrangement is used to minimize mutual coupling, it is difficult to achieve complete isolation between radiating elements in practice. Some level of coupling might still exist, leading to deviations from the simulated results. Also, in simulations, certain assumptions and simplifications are made to facilitate computation. These assumptions might not fully capture all real-world complexities, leading to discrepancies between the simulated and measured results.

### 7.2. 2D Radiation Patterns

**Figure 14:**
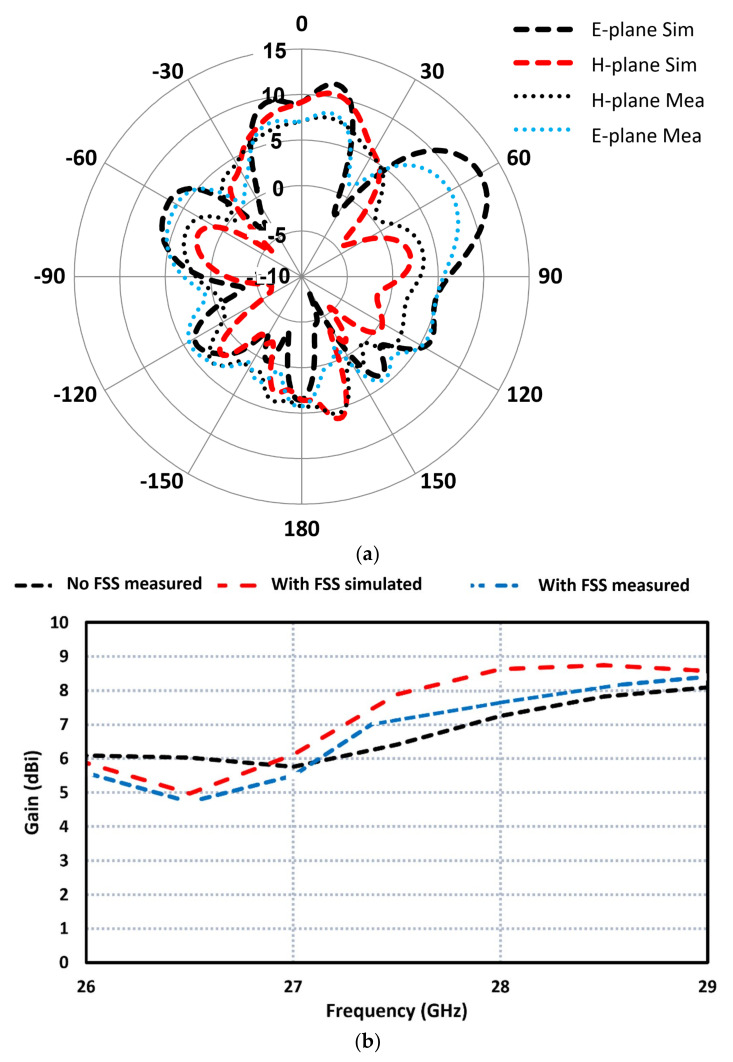
Measured and simulated plots of (**a**) radiation patterns and (**b**) gain of the FSS-based MIMO antenna array.

The simulated and measured polar plots of FSS-based MIMO antenna at 28 GHz are shown in Figure 14a. The radiation patterns are shown for both E-plane and H-plane. The radiation from the MIMO antenna is directional and perpendicular to the plain of the antenna. The simulated gain at an angle of 0 degrees in the E- and H-planes is 8.4 dBi; however, the measured gain at this angle is 7.5 dBi. The measured radiation in the E- and H-planes is virtually identical over the 3-dB beamwidth of the MIMO antenna centered at 0 degrees. This confirms that circular polarization is maintained over the antenna’s 3-dB beamwidth. Figure 14b shows how the gain of the array was affected by the inclusion of the FSS. Without FSS, the measured gain was 7.2 dBi, and with FSS, the gain increased to 8.6 dBi. The radiation efficiency of the MIMO antenna array at 28 GHz was measured to be 85%, and across its operational band, the efficiency was 80%. The discrepancy in the simulated and measured results as mentioned previously is attributed to manufacturing tolerances, unwanted antenna coupling, and inaccurate simulation models.

## 8. MIMO Performance Parameters

### 8.1. Envelope Correlation Coefficient

**Figure 15:**
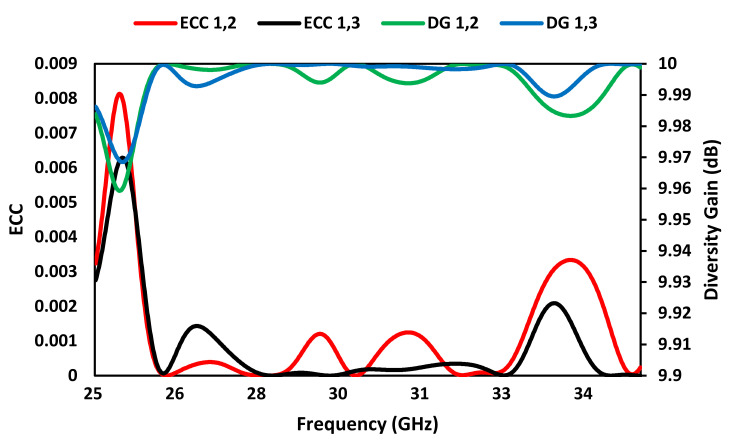
ECC and DG of the proposed FSS-based MIMO antenna array.

The Envelope Correlation Coefficient (ECC) is a metric used to quantify the correlation between two antennas in an MIMO system. A high correlation between antennas can limit the benefits of spatial diversity and reduce the potential capacity gain that MIMO offers. On the other hand, a low correlation is desirable because it allows the MIMO system to achieve higher data rates and improved link reliability. The value of ECC should be less than 0.5 to minimize coupling effects. The value of ECC can be estimated using the relation in [17].

(2) $$ECC=\frac{|\iint 4\pi (M_{i})(\theta ,\phi ))\times (\;M_{j}(\theta ,\phi ))d\Omega {|}^{2}}{\iint 4\pi |(M_{i}\left(\theta ,\phi \right)){|}^{2}d\Omega \iint 4\pi |(M_{j}\left(\theta ,\phi \right)){|}^{2}d\Omega }\;\;\;$$

where *M_i_* (θ,ϕ) and *M_j_* (θ,ϕ)  represent the radiation patterns when antennas *i* and *j* are excited, and the term Ω denotes the solid angle. The ECC of the proposed FSS-based MIMO antenna array as a function of frequency in Figure 15 shows that it is less than 0.002 in the 5G operating band at 28 GHz between 26.5 GHz and 29.5 GHz.

### 8.2. Diversity Gain

Diversity gain (DG) is the improvement in signal quality and system performance achieved by utilizing multiple antennas at both the transmitter and receiver. The diversity gain of MIMO antenna array can be calculated from the ECC using the following relation [17]:

(3) $$DG=10\times \sqrt{\left(1-ECC\right)}$$

The diversity gain of the proposed MIMO array in Figure 15 shows that it is better than 9.99 dB in the 5G operating band at 28 GHz between 26.5 GHz and 29.5 GHz.

The performance of the proposed MIMO antenna array is compared in Table 4 with other MIMO arrays reported in the literature. Compared to other four-port devices, the proposed MIMO array with FSS exhibits a higher gain and has the smallest ECC. The dimensions of the proposed MIMO array with FSS are comparable to other MIMO arrays cited in the table.

## 9. Conclusions

In this article, we have demonstrated that mutual coupling between neighboring antennas in an MIMO array can be reduced by spatially arranging the individual antennas orthogonally relative to each other. It is also shown that the impedance bandwidth of the antenna can be widened by defecting the ground plane with a rectangular slot. Moreover, it is shown that the gain of the MIMO array can be enhanced by locating a frequency-selective surface (FSS) over the MIMO array. The FSS unit cell was designed to allow the frequency band at 28 GHz. The gap between the MIMO array and FSS was adjusted to one-half guided wavelength to ensure constructive interference between the forward and FSS reflected electromagnetic waves. The frequency-selective surface consisted of a 7 × 7 matrix of FSS unit cells. The measured results show that the gain of the MIMO array is increased by including the FSS. The proposed FSS-based MIMO array has an impedance bandwidth between 27.2 GHz and 29 GHz with a total efficiency of 86% at 28 GHz. Moreover, its ECC is less than 0.002 and its diversity gain is better than 9.99 dB in the 5G operating band centered at 28 GHz between 26.5 GHz and 29.5 GHz.

## Figures and Tables

**Table 1:** Design parameters of modified circular patch antenna.

Symbol	Dimensions (mm)
L_s_	18
L_g_	18
L_f_	6
W_f_	1.5
R	6
R1	2
Lc	13.9
Wc	2.4
W_s_	19
W_g_	19

**Table 2:** Dimensions of ground plane rectangular slot used in parametric study.

Slot Length (Lc) (mm)	Slot Width (Wc) (mm)	Freq. Range (GHz)	Impedance BW (GHz)
5	1	26.6–27.6	1
7	1.5	26.4–27.2	0.8
9.5	2	33.2–34	0.8
13.9	2.4	27–29	2

**Table 3:** Design parameters of the proposed FSS unit cell.

Parameters	Symbols	Value (mm)
Substrate length and width	Ls = Ws	3.7
Gap one	g1	0.24
Gap two	g2	0.25
Gap three	g3	0.27
Length of FSS	Lm	45
Width of FSS	Wm	45

**Table 4:** Comparison with other reported MIMO antenna arrays.

Ref	*f* (GHz)	Ports	Dimension in (mm)	Min. Isolation (dB)	Gain (dBi)	ECC	Techniques
[9]	28	2	30 × 15 × 0.25	35.8	5.42	<0.005	DGS
[13]	27	4	30 × 30 × 1.575	30	7.1	<0.005	DGS
[14]	28	4	30 × 35 × 0.76	17	8.3	<0.010	DGS
[8]	28	2	15 × 25 × 0.203	30	5.8	<0.005	DGS
[15]	28	4	30 × 30 × 0.787	29	6.1	<0.160	DGS
[16]	28	4	20 × 40 × 1.6	29.34	7	<0.010	DRA
[17]	27	4	30 × 28 × 0.508	24	6.22	<0.050	DGS
[18]	28	2	18 × 36 × 0.8	64	8.75	<0.050	SRR/DGS
Proposed MIMO without FSS	28	4	38 × 36 × 0.8	26.31	7.2	<0.002	DGS
Proposed MIMO with FSS	28	4	45 × 45 × 0.8	23.31	8.6	<0.002	FSS/DGS

## Data Availability

All data generated or analyzed during this study are included in this article. All of the figures, materials, and data within the manuscript are original and owned by authors.
